# The utility of software-detected non-invasive tear break-up in comparison to fluorescein tear break-up measurements

**DOI:** 10.3389/fmed.2024.1351013

**Published:** 2024-07-04

**Authors:** Stephen C. Pflugfelder, Yasushi Kikukawa, Shin Tanaka, Takuya Kosugi

**Affiliations:** ^1^Department of Ophthalmology, Baylor College of Medicine, Houston, TX, United States; ^2^Kowa Company, Ltd., Tokyo, Japan; ^3^Kowa Ophthalmic Research Laboratories, Kowa Research Institute Inc., Boston, MA, United States

**Keywords:** dry eye, tear stability, tear break-up time, interferometry, artificial intelligence

## Abstract

**Purpose:**

The purpose of this study is to characterize and discuss the difference between software-detected non-invasive tear break-up time (NIBUT) and the traditional clinical method of fluorescein break-up time (FBUT).

**Methods:**

Tear interferometry with the KOWA DR-1α (Kowa, Japan) and a standardized comprehensive ocular surface/tear evaluation were performed in 307 eyes. Software-detected NIBUT in the KOWA DR-1α images and the investigator-detected FBUT were compared.

**Results:**

Software-detected NIBUT was significantly shorter than investigator-measured FBUT. NIBUT was 3.1 ± 2.5 s (mean ± SD), whereas FBUT was 4.8 ± 3.0 s. This difference was due to three different patterns or conditions: a spot break immediately after eyelid opening, moderate to severe keratitis sicca, and epithelial basement membrane corneal dystrophy (EBMD). In these cases, rapid tear film disruption was not captured by FBUT. A spot break immediately after eye opening that rapidly disappears was observed with conjunctivochalasis. This type of break-up may be difficult to detect using fluorescein because the human eye cannot catch such rapid blinks or post-blink events. In the second group with severe corneal epithelial disease, break-up may occur over the entire corneal surface upon eye opening, and distinct fluorescein tear break-up may not be identified because of poor dye dilution or spread over the corneal surface, whereas the non-invasive break-up is not solution-dependent, and the software can detect a distinct appearance. In the third group with EBMD, it is possible that focal break-up in the fluorescein pattern over the epithelial elevations, which might be missed visually, can be detected by software in video images.

**Conclusion:**

We found that software-detected NIBUT is more sensitive in detecting tear break-up, can identify certain tear film disruptions that are missed by traditional FBUT, and may be more useful in distinguishing certain tear disorders.

## 1 Introduction

Tear instability is the defining feature of dry eye disease (1). Traditionally, this has been visually detected as discontinuities in the fluorescein-stained tear film, termed fluorescein break-up time (FBUT). Non-invasive methods have been developed to detect break-up in an image (2). The KOWA DR-1α uses interferometry to image the tear lipid layer. Break-up in these interferometric images can be detected visually, and software has been developed and fine-tuned to detect and measure break-up (3). Dry eye is a heterogeneous disease that includes conditions with reduced tear volume and conditions with adequate tear volume that have alterations of the corneal or conjunctival surfaces, such as corneal epithelial basement membrane disease (EBMD) or conjunctivochalasis, which can mechanically disrupt tear distribution and stability (4). In eyes with diffuse EBMD, the initial site of fluorescein tear break-up may be missed, or tear break-up may occur simultaneously in several areas. Furthermore, in eyes with severe aqueous deficiency, instilled fluorescein dye may not adequately spread or mix with the tear film to allow proper visualization. Additionally, fluorescein may self-quench due to poor dilution (5). Consequently, FBUT may be difficult to evaluate or may not be accurately detected in these conditions. The purpose of this study is to compare the utility of software-detected non-invasive tear break-up in KOWA DR-1α interferometry images to FBUT measurements for a variety of tear disorders, with particular attention to these conditions.

## 2 Patients and methods

This study was approved by the Baylor College of Medicine Institutional Review Board (IRB; Protocol Number H-51925), and all research adhered to the tenets of the Declaration of Helsinki. A retrospective chart review was conducted on all patients who received a comprehensive ocular surface examination for dry eye at the Alkek Eye Center from 2019 to 2022. Patients who had keratoneuralgia or non-tear film-related eye discomfort were excluded from the study.

All patients underwent a standard panel of tear film and ocular surface tests in the following order: a Symptom Assessment in Dry Eye (SANDE) symptom questionnaire, interferometric analysis of tear stability with the KOWA DR-1α, optical coherence tomography measurement of tear meniscus height (Avanti, Optovue, CA), biomicroscopic examination, fluorescein tear break-up time (FBUT), cornea fluorescein staining, and conjunctival lissamine green staining. These tests were performed according to previously reported methods (6). The severity of the ocular surface disease was graded 0–3 using previously reported severity criteria (7, 8). Dry eye was classified as aqueous deficiency, meibomian gland disease, conjunctivochalasis, or other categories based on previously published criteria (6, 9).

FBUT was measured by applying a drop of fluorescein dye to the lower tarsal conjunctiva using a fluorescein strip (BioGlo, HUB, Rancho Cucamonga, CA) wetted with a drop of preservative-free saline (Addipak, Teleflex, Research Triangle Park, NC). The patient was instructed to blink twice to distribute the fluorescein, then blink and keep the eye open while the time elapsed from the last blink to the appearance of the first break in the continuous layer of fluorescein, observed using a slit-lamp (Haag-Streit, Haag Streit, Koeniz, Switzerland) under cobalt blue light, was measured as the FBUT using a stopwatch.

The KOWA DR-1α instrument was calibrated daily using a standardized procedure. The light intensity control knob was set to the 10 o’clock position and the observation area switching knob was set to wide. The examination was performed for 30 s in each eye, and the video data were saved.

For a software analysis of NIBUT, an image classification model was developed to detect tear break-up non-invasively (3) by modifying the ResNet50 model. It was pre-trained on the ImageNet dataset to build a convolutional neural network model for detecting the characteristics of tear interferometric images by performing transfer learning on images extracted from videos recorded by the KOWA DR-1α. The model can detect three classes of tear break-up based on their shape: area break, spot break, and line break.

The software used in the analysis of the KOWA DR-1α was also used to detect inter-blink intervals and tear break-up. NIBUT using the software was measured as the time elapsed between the last blink and the detection of the first break-up. In our previous study, it was verified that there was a good correlation between investigator-visually detected and software-detected tear break-up time in the KOWA DR-1α interferometric fringe images (10). The investigator observed and checked the video frame-by-frame to identify the initial break in order to avoid overlooking and detecting errors.

### 2.1 Statistical analysis

Statistical tests were carried out using a statistical programming language R (version 3.6.1, The R Foundation for Statistical Computing, Vienna, Austria). Software-detected NIBUT and visually measured FBUT were compared using a linear mixed model where the random effect was patients.

## 3 Results

A total of 307 examinations were performed on 204 patients over a 3-year period. Tear and ocular surface clinical data are shown in Table 1.

**Table 1:** Summary of clinical data.

Age (yrs)	Sex	Eyes	SANDE	FBUT seconds	NIBUT seconds	Cornea FL staining	Conjunctiva LG staining	Severity (0–3)
61.6 ± 14.5	F:163	R *n* = 158		4.9 ± 3.0	3.3 ± 2.7	2.6 ± 3.4	1.7 ± 1.9	1.8 ± 0.9
	M:41	L *n* = 149		4.7 ± 2.9	2.9 ± 2.2	2.8 ± 3.6	1.9 ± 2.0	1.9 ± 0.9
		Total *n* = 307	76 ± 27	4.8 ± 3.0	3.1 ± 2.5	2.7 ± 3.5	1.8 ± 2.0	1.8 ± 0.9

Data reported as mean ± standard deviation. SANDE, Symptom Assessment in Dry Eye questionnaire (scale 0–100 mm); FBUT, fluorescein tear break-up time; NIBUT, non-invasive tear break-up time. FL, fluorescein dye; LG, lissamine green dye, severity (0–3) based on previously reported criteria.

### 3.1 Comparison of FBUT and NIBUT in the entire group

Software-detected NIBUT was significantly shorter than investigator-measured FBUT. NIBUT was 3.1 ± 2.5 s (mean ± SD), whereas FBUT was 4.8 ± 3.0 s (*p* < 0.001). This difference was due to three different patterns or conditions: spot break immediately after eyelid opening, moderate to severe keratitis sicca, and epithelial basement membrane corneal dystrophy (EBMD).

### 3.2 Spot break immediately after eyelid opening

**Figure 1:**
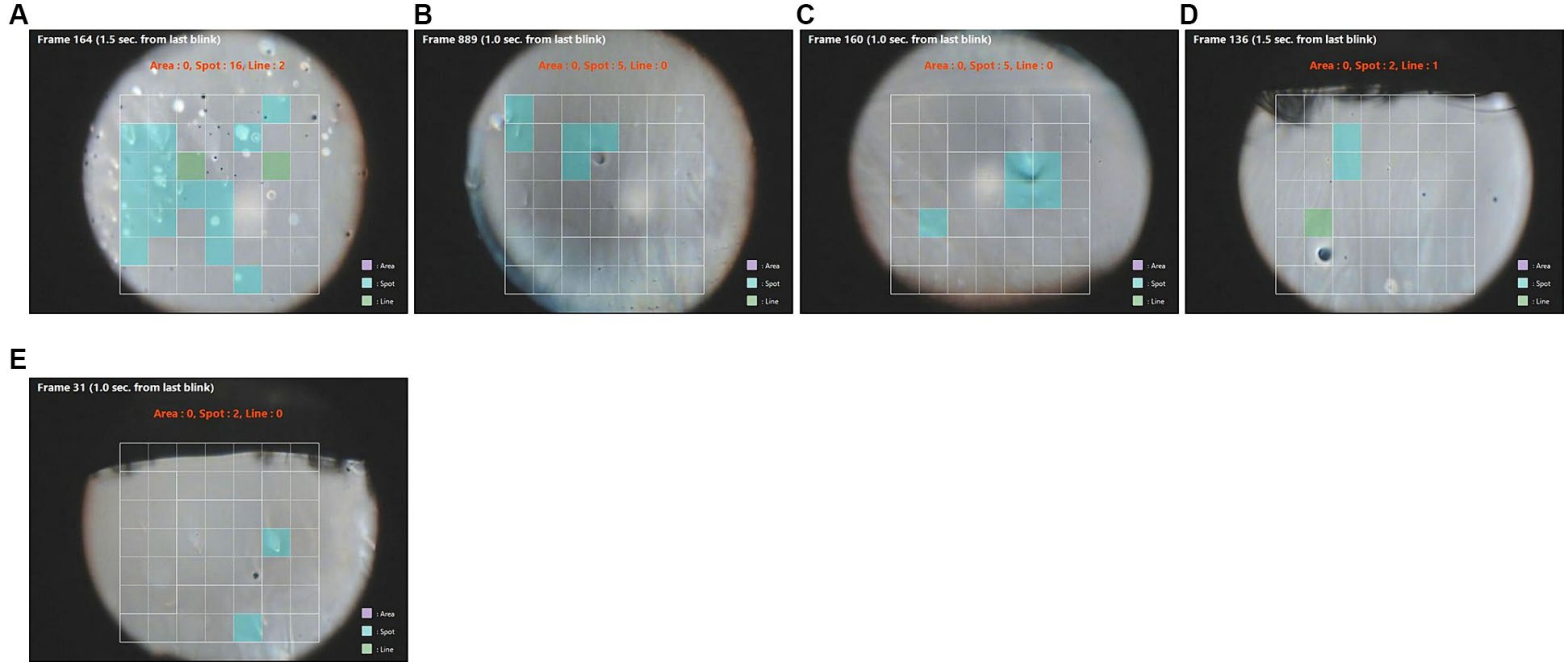
**(A–E)** Images of spot break-up after a blink (blue squares) in five different patients. NIBUT was more rapid than FBUT in these eyes (Table 2). Green squares are areas of line break-up.

This type of break appeared as a bubble in the precorneal tear film. Spot break was detected by software in 55 eyes, and among these, 10 eyes with this type of break-up pattern had conjunctivochalasis (CCh; Table 2). Of these 10 eyes, we found that five eyes had a larger difference between FBUT and NIBUT (Table 3; Figure 1). In fluorescein break-up, a similar ratio of spot breaks to total breaks was noted in eyes with CCh (28%) compared to the ratio in all cases (31%). In contrast, software-detected NIBUT revealed that the ratio of spot breaks to total breaks was higher in CCh than in all cases (28 vs. 18%, respectively, Table 4). These data indicate that spot breaks are more likely to be detected by the software in eyes with CCh than other tear dysfunction conditions compared to FBUT.

**Table 2:** Association of spot break with conjunctivochalasis.

Classification	Break-up detected by software [eyes]	Spot break detected [eyes]	Ratio [%] (spot/total break)
All	307	55	18
CCh	36	10	28

Non-invasive tear break-up was detected by software in 307 eyes in the entire group, which includes 36 eyes with conjunctivochalasis (CCh).

**Table 3:** Spot break immediately after eyelid opening.

ID	Eye	Classification	NIBUT[s]	NIBUT frame	FBUT[s]
86	L	CCh	1.5	164	4
104	L	CCh	1.0	889	9
104	R	CCh	1.0	160	9
108	L	CCh	1.5	136	4
220	R	CCh	1.0	31	7

NIBUT, non-invasive tear break-up time in seconds (the video frame number) where break-up is detected is provided; FBUT, fluorescein tear break-up time in seconds.

**Table 4:** Ratios of spot break in all eyes and eyes with conjunctivochalasis.

BUT	FBUT	NIBUT
Spot break detected [all eyes] (Total: 307 eyes)	96 eyes (31%)	55 eyes (18%)
Spot break detected in CCh [eyes] (Total: 36 eyes)	10 eyes (28%)	10 eyes (28%)

### 3.3 Severe keratitis sicca

**Figure 2:**
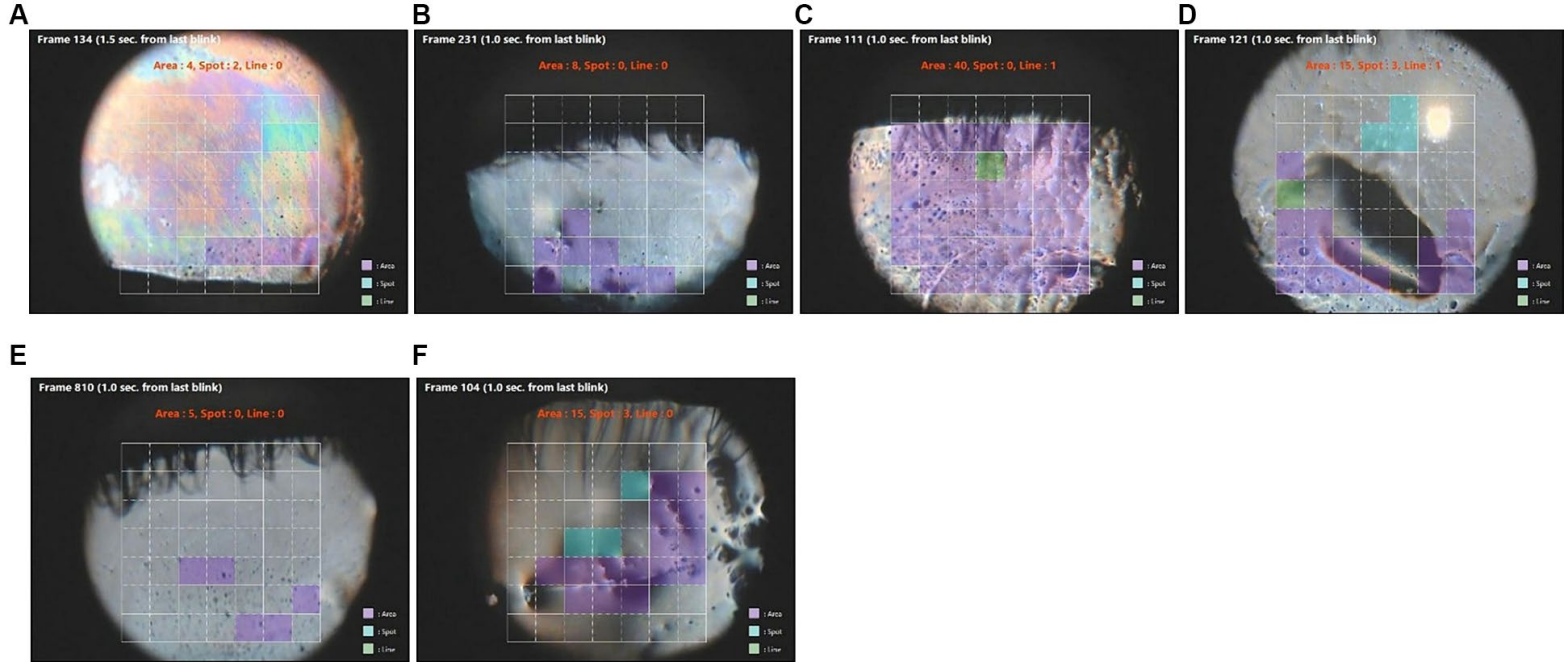
Images of rapid area tear break-up (purple squares) detected in video images in eyes with severe keratitis sicca (fluorescein staining scores >7). **(A–F)** correspond to the ID numbers in Table 5 (*A* = 8, *B* = 29, *C* = 69, *D* = 190, *E* = 231. *F* = 278). Green squares are areas of line break-up and blue squares are areas of spot break-up.

In eyes with severe corneal fluorescein staining (scores >7), diffuse area break-up occurring over the entire corneal surface was detected in the interferometric images by software earlier than fluorescein break-up in seven eyes (Table 5; Figure 2). This group included eyes with low (<273 um) or elevated (>345 um) tear meniscus heights (Table 5). A higher ratio of area break was noted in eyes with corneal fluorescein staining scores of >7 overall compared to all cases (Table 6). Area breaks were detected in 77% (24/31) of eyes with corneal fluorescein staining scores >7, whereas they were detected in only 22% (68/307) of break-ups in all eyes.

**Table 5:** Area break-up in eyes with severe keratitis sicca.

ID	Eye	Cornea FL staining	NIBUT[s]	NIBUT frame	FBUT[s]	TMH [um]
8	R	9	1.5	134	3.0	273
29	R	10	1.0	231	3.0	434
68	L	12	1.0	111	2.0	1,500
190	L	12	1.0	121	4.0	186
231	R	15	1.0	810	2.0	345
278	L	12	1.0	104	2.0	761

**Table 6:** Non-invasive area break-up.

Break-up	Eyes	Cornea FL staining >7	Cornea FL staining ≤7
All	307	31 eyes (10%)	276 eyes (90%)
Area break-up	68	24 eyes (35%)	44 eyes (65%)

Non-invasive tear break-up was detected by software in 307 eyes in the entire group and area break-up was noted in 68 of these eyes. Fluorescein (FL) staining scores range from 0 to 15.

### 3.4 Epithelial basement membrane disease

**Figure 3:**
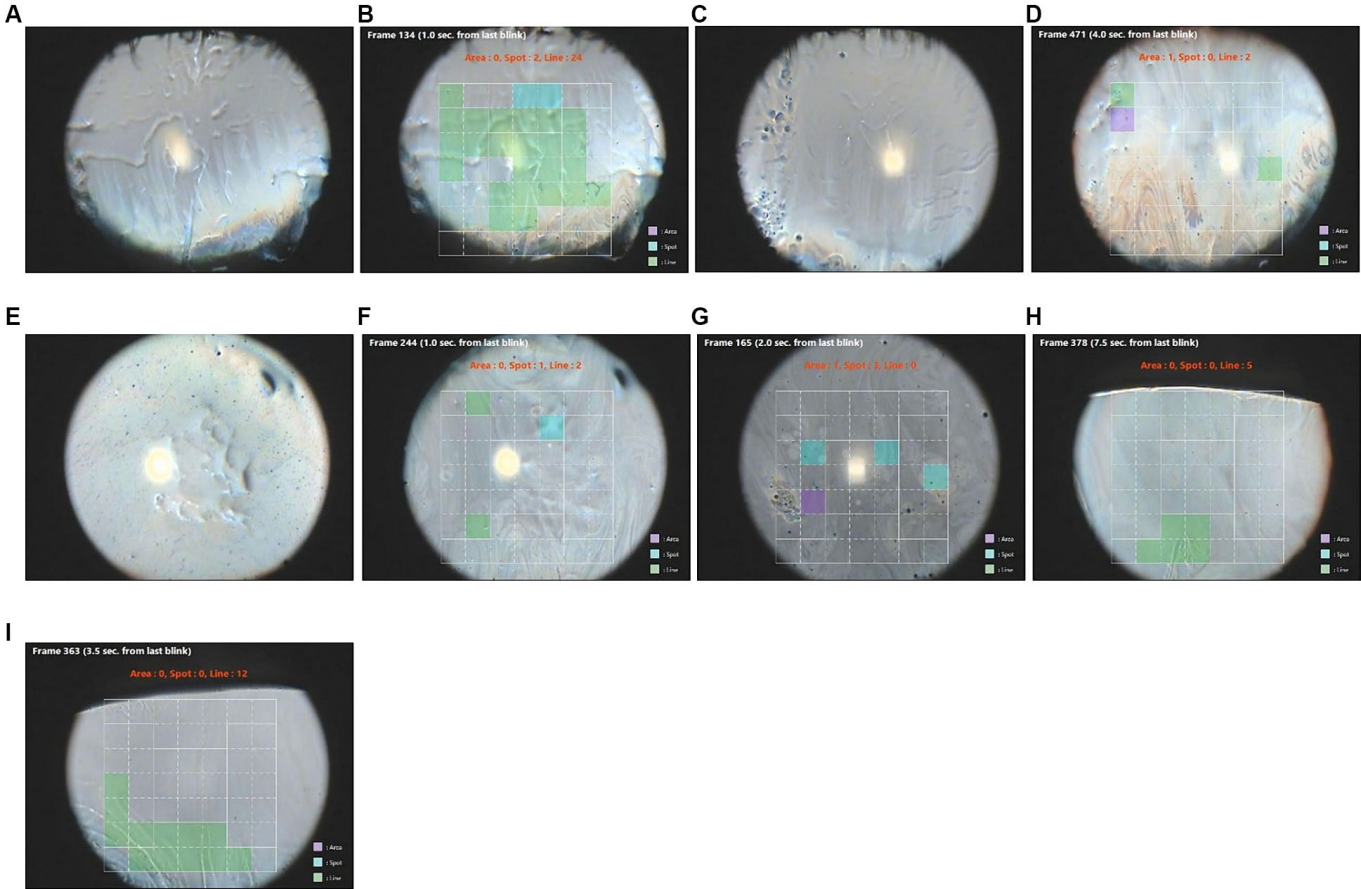
Images of tear break-up in eyes with epithelial basement membrane disease (EBMD). Green squares are areas of line break-up, blue squares are areas of spot break-up, and purple squares are areas of area break-up. **(A)** ID 100 R eye before blinking, **(B)** ID 100 R eye break-up detected in colored squares, **(C)** ID 100 L eye before blinking, **(D)** ID 100 L eye break-up detected in colored squares, **(E)** ID 114 R eye before blinking, **(F)** ID 114 R eye break-up detected in colored squares, **(G)** ID 114 L eye break-up detected in colored squares, **(H)** ID 212 R eye break-up detected in colored squares, and **(I)** ID 212 L break-up detected in colored squares.

NIBUT in the KOWA DR-1α image was more rapid than FBUT in four eyes with epithelial basement membrane disease (EBMD) associated with either ATD or CCh (Table 7). This suggests that there is a tear break-up over these basement membrane deposits that can be software-detected but not visually with fluorescein. The basement membrane deposits were visible prior to the blink in three of these eyes (Table 7 and Figures 3A,C,E).

**Table 7:** Tear break-up in eyes with epithelial basement membrane disease.

ID	Eye	Classification	NIBUT[s]	NIBUT frame	FBUT[s]	Presence of BM deposits
100	R	ATD/EBMD	1.0	134	12	Yes
100	L	ATD/EBMD	4.0	471	11	Yes
114	R	CCh/EBMD	1.0	244	4	Yes
114	L	CCh/EBMD	2.0	165	4	No
212	R	EBMD	7.5	378	3	No
212	L	EBMD	3.5	363	3	No

NIBUT, non-invasive tear break-up time in seconds (the video frame number where break-up is detected is provided); FBUT, fluorescein tear break-up time in seconds; BM, basement membrane; ATD, aqueous tear deficiency; EBMD, epithelial basement membrane disease; CCh, conjunctivochalasis.

## 4 Discussion

This study evaluated the utility of measuring NIBUT using the KOWA DR-1α. Software-measured NIBUT was significantly faster than investigator-measured FBUT. Upon deeper analysis, the difference was found to be largely attributed to three conditions: CCh, severe keratitis sicca defined by high corneal fluorescein staining scores, and certain eyes with EBMD. These findings suggest that software may be able to detect discontinuities in interferometric images of the tear film earlier than they can be detected visually in the fluorescein-stained tear film. This suggests that software-detected NIBUT may be more sensitive in identifying an unstable tear film.

Different patterns of fluorescein tear break-up have been identified, and certain patterns have a reported association with specific disorders (e.g., area break-up with severe aqueous deficiency) (11). Our findings with the KOWA DR-1α software detection would support this concept. Rapid area break-up was found with increased frequency in eyes with severe keratitis sicca, and spot break-up immediately after eye opening was associated with CCh.

A previous study using the KOWA DR-1α has reported that NIBUT was longer than FBUT (12). In this study, the reasons why NIBUT is more sensitive than the fluorescein method in detecting tear break in these conditions remain to be determined, but it could be related to factors that have been modeled to affect fluorescein tear break-up, including tear volume, thickness, osmolarity, and tear spread (13). Quenching of fluorescence has been observed in eyes with low tear volume (5). Changes in the corneal epithelium in severe KCS, such as reduced production of membrane mucins and increased expression of cornified envelope precursors, may affect tear diffusion and adherence of tear fluid to the corneal epithelium (14). Lid parallel folds in conjunctivochalasis mechanically disrupt the tear meniscus, which can sequester tears and impede the mixing of fluorescein. These factors may interfere with the detection of fluorescein break-up (9, 15). Additionally, the rapid spot break in CCh occurring as the eyelid rises during a blink may have been overlooked in the fluorescein method. This is because spot breaks might be small (less than a few percent of the observed area) or are likely to disappear and/or move to various portions of the cornea during the upward movement of aqueous tear fluid after the eye opening, which can be overlooked. Evaluation of the tear lipid layer in interferometric images may be more sensitive in detecting tear break-up than fluorescein in some eyes with EBMD. It is possible that fluorescein does not break-up over these deposits, but software can detect a distinct interferometric appearance. It is interesting that all of the eyes with EBMD that had more rapid NIBUT had associated CCh or ATD.

In summary, software-detected non-invasive tear break-up appears to be more sensitive in detecting tear break-up in certain conditions. Software-detected NIBUT may prove to be valuable in the diagnostic classification of tear disorders, and the ability to detect break-up time and patterns may improve when the software is trained with a larger library of images containing a variety of tear dysfunction conditions.

## Data availability statement

The raw data supporting the conclusions of this article will be made available by the authors, without undue reservation.

## Ethics statement

The studies involving humans were approved by Baylor College of Medicine Institutional Review Board (IRB; Protocol number H-51925). The studies were conducted in accordance with the local legislation and institutional requirements. The ethics committee/institutional review board waived the requirement of written informed consent for participation from the participants or the participants’ legal guardians/next of kin because this is a retrospective data review.

## Author contributions

SP: Formal analysis, Investigation, Writing – original draft. YK: Formal analysis, Software, Writing – original draft. ST: Formal analysis, Software, Writing – review & editing. TK: Formal analysis, Software, Writing – review & editing.
